# Jatropha half-sib family selection with high adaptability and genotypic stability

**DOI:** 10.1371/journal.pone.0199880

**Published:** 2018-07-12

**Authors:** Leonardo de Azevedo Peixoto, Paulo Eduardo Teodoro, Lidiane Aparecida Silva, Erina Vitório Rodrigues, Bruno Galvêas Laviola, Leonardo Lopes Bhering

**Affiliations:** 1 Universidade Federal de Viçosa, Avenida Peter Henry Rolfs, s/n, Campus Universitário, Viçosa, Minas Gerais, Brasil; 2 Embrapa Agroenergia, Parque Estação Biológica (PqEB), Asa Norte, Brasília, Federal District, Brazil; College of Agricultural Sciences, UNITED STATES

## Abstract

Jatropha (*Jatropha curcas*) has become one of the most important species for producing biofuels. Currently, Genotype x Environment (GxE) interaction is the biggest challenge that breeders should solve to increase the section accuracy in the plant breeding. Therefore, the objectives in this study were to estimate the parameters in the 180 half-sib families in Jatropha evaluated for five production years, to verify the significance of the GxE interaction variance, to evaluate the adaptability and stability for each family based on three prediction methods, to select superior half-sib families based on the adaptability and stability analyses, and to predict the accuracy for the sixth production year. Jatropha half-sib families were classified and selected using the follow adaptability and stability methods: linear regression, bi-segmented linear regression and mixed models concepts called harmonic mean of the relative performance of genetic values (HMRPGV). The prediction accuracy was estimated by the Pearson correlation between the predicted genetic values by adaptability and stability methods and the phenotypic value in the sixth production year. In result, most half-sib families were classified as general adaptability and general stability for the evaluated traits. The selection gain obtained via HMRPGV was higher than other methods. The prediction accuracy for the sixth production year was 0.45. Therefore, HMRPGV is efficient to maximize the genetic gain, and it can be a useful strategy to select genotype with high adaptability and stability in Jatropha breeding as well as other species that should be evaluated for many years to take a suitable selection accuracy.

## Introduction

Jatropha (*Jatropha curcas* L.) is a perennial plant monoecious, and belongs to *Euforbiáceae* family. This species has several used, such as living fence, phytoremediation, and medicinal purposes [1]. However, due to the worldwide corner about climate change Jatropha has become to be important to biofuel production, because it presents high oil content in the seeds [2], oil/grain ratio superior than traditional oleaginous, for example, soybean [3]. Despite that Jatropha is a exotic culture, it has been cultivated in almost all Brazilian regions, in fact, it a culture adapted for different environments [4–6].

The effects of Genotype x Environment (GxE) interaction has been the most changeling factor in plant breeding because it makes the varieties recommendation more difficult. However, it is necessary to evaluate the GxE interaction and uses it as an advantage to recommend genotypes for different soil and climate condition. Besides that it is important to evaluate this magnitude by adaptability and stability studies [7]. Several methods can be found on the literature such as based on linear regression [8], bi-segmented linear regression [9], and most recently mixed models concepts called harmonic mean of the relative performance of genetic values (HMRPGV). It is worth mentioning that the uses of the correct methods will influence directly the selection of superior genotypes.

Currently, Eberhart and Russell [8] method is the most used method to study adaptability and stability due to it is simpler to calculate parameters and to understand the results. It considers that genotype performance in each environment is estimated by linear regression analysis based on their phenotypic values in function of environmental gradient [7]. On other words, adaptability is evaluated by linear regression analysis, which each genotype is fitted by one linear regression equation. Whereas stability is estimated by the sum of deviations calculated for each environment.

Cruz *et al*. [9] presented a modification for the Eberhart and Russell [8] making a simplification for estimating parameters and calculating sum of squares, and consequently having statistics proprieties more suitable for breeding purpose. This method bases on the bi-segmented linear regression, which three adaptability parameters are estimated: mean, linear performance for unfavorable environments, and linear performance for favorable environment. Based on this method, it is possible to select superior genotype for general performance as well as for favorable and unfavorable environment. However, this method has as advantages to analyze more parameters.

Although, there were a huge upgrade on GxE interaction methodologies, from the traditional analysis based on bi-segmented linear regression, these methods have some limitations. It is possible to point out the impossibility to perform these methods for unbalanced experiment, unbalanced orthogonal array, and experiments evaluated in many environments with heterogeneity variance. Situations cited above is very common in plant breeding, especially in forest breeding. In addition, these methods usually assigned genotype effects as fixed, and this is a disadvantage as well as it is not true when the objective is to estimated variance components and genetic parameters based on these methods [10].

Regarding de Resende [11] proposed a methodology which it makes possible to select superior genotypes with general adaptability and stability based on mixed models (HMRPGV). This method allows to select genotypes for the three features (general mean, adaptability and stability) simultaneously and, it present several advantages: genotype effects are assigned as random effect, and therefore, this method estimates the genotypic adaptability and stability, instead of the phenotypic adaptability and stability; this method can be used for unbalanced experiment and unbalanced orthogonal array; it is possible to work with heterogeneity variance and correlated errors within block or environment; it can be apply with any environment numbers; it makes possible to consider the adaptability and stability to select superior genotypes within progenies; it does not depend on estimation or interpretation of other parameters such as regression coefficient; it allows to eliminate the GxE interaction error, due to it considers the heritability for this effect; it generates results on the same magnitude of the phenotypic values for the trait; and it estimates the genetic gain for the three features simultaneously [10].

For all reasons discussed above the objectives for this study were: i) to estimate the parameters in the 180 half-sib families in Jatropha evaluated for five production years; ii) to verify the significance of the GxE interaction variance; iii) to estimate the adaptability and stability for each family based on three prediction methods; iv) to select superior half-sib families based on the adaptability and stability analyses; and v) to estimate the prediction accuracy for the sixth production year.

## Material and methods

### Experimental design

The experiment was installed in the experimental area of Embrapa Cerrados, in Planaltina, DF (lat. 15°35'30''S, long. 47 °42'30''W, at 1,007 m asl) in November 2008. The climate is tropical with dry winter and rainy summer (Aw), according to the Köppen classification, with average annual temperature of 22°C, relative humidity of 73%, and average annual rainfall of 1,100 mm. The predominant soil at the site was classified as Red Latosol, with a high clay content.

180 Jatropha half-sib families were evaluated using a randomized block design with two replications and five plants per plot, spaced 4 x 2 m apart. The parents used for the development of half-sib families had genetic variability for the characteristics of field production (PROD) and weight of 100 seeds (W100S) and they were harvested randomly around Brazilian Jatropha field. Management practices were based on Dias *et al*. [12], adapted according to the results of studies on Jatropha in Brazil and in the world. The half-sib families were evaluated over five crop years (2010 to 2014) for PROD and four crop years (2010 to 2013) for W100S.

### Statistical analysis

Firstly, analysis of variance and Deviance analysis was performed for each year following the model:

$$Y_{ij}=\mu +B_{j}+G_{i}+\varepsilon_{ij}$$

In which: Y_ij_ is the phenotypic value of the i^th^ family in the j^th^ block; μ is the general mean; B_j_ is the j^th^ block effect assigned as fixed effect; G_i_ is the i^th^ family effect assigned as random effect; ε_ij_ is the random error.

Secondly, analysis of variance was performed considering all years based on the model below:

$$Y_{ijk}=\mu +B_{k}+G_{i}+E_{j}+{GE}_{ij}+\varepsilon_{ij}$$

In which Y_ijk_ is the phenotypic value of the i^th^ family evaluated in the j^th^ year in the k^th^ block; B_j_ is the j^th^ block effect assigned as fixed effect; G_i_ is the i^th^ family effect assigned as random effect; E_j_ is the j^th^ year effect assigned as fixed effect; GE_(ij)_ is the GxE interaction effect assigned as random effect; ε_ij_ is the random error.

Variance components were estimated by least squares method:

$${\hat{\sigma }}_{g}^{2}=\frac{{MS}_{g}-{MS}_{r}}{er}$$

in which: ${\hat{\sigma }}_{g}^{2}$ is the genotypic variance among half-sib families; *MS*_*g*_ is the mean square of the half-sib families; *MS*_*r*_ is the mean square of the residual; *e* is the number of environments (years); *r* is the number of replication (Blocks).

$${\hat{\sigma }}_{gxe}^{2}=\frac{{(MS}_{gxe}-{MS}_{r})(e-1)}{er}$$

in which: ${\hat{\sigma }}_{gxe}^{2}$ is the GxE interaction variance GxE; *MS*_*gxe*_ is the mean square of the GxE interaction.

$${\hat{\sigma }}^{2}={MS}_{r}$$

in which;${\hat{\sigma }}^{2}$ is the residual variance.

$${\hat{\sigma }}_{f}^{2}=\frac{{MS}_{g}}{er}$$

in which; ${\hat{\sigma }}_{f}^{2}$ in the phenotypic variance among half-sib families.

$${\hat{h}}^{2}=\frac{{\hat{\sigma }}_{g}^{2}}{{\hat{\sigma }}_{f}^{2}}$$

in which ${\hat{h}}^{2}$ is the heritability among half-sib families.

Subsequently, the data was analyzed by the restricted maximum likelihood (REML) following the model:

Y=Xb+Zg+Xc+ε

in which: Y is the phenotypic values vector; b is the block effects vector assigned as fixed effect; g is the genotypic family effect vector assigned as random effect; c is the GxE interaction effect vector assigned as random effect; ε is the random error vector; X, Z, and W are the incidence matrix for the b, g, and c effects, respectively.

Distribution and structure of the variance and mean vector were respectively:

$$E\left[\begin{array}{c} Y\\ g\\ \begin{array}{c} c\\ \varepsilon \end{array} \end{array}]=[\begin{array}{c} Xb\\ 0\\ \begin{array}{c} 0\\ 0 \end{array} \end{array}\right];$$

$$Var\left[\begin{array}{c} g\\ c\\ e \end{array}\right]=\left[\begin{array}{ccc} I\sigma_{g}^{2} & 0 & 0\\ 0 & I\sigma_{gxe}^{2} & 0\\ 0 & 0 & I\sigma_{e}^{2} \end{array}\right]$$

Aiming to estimate the fixed effects and to predict the random effects mixed models equation were performed as described below:

$$\left[\begin{array}{ccc} X\prime X & X\prime Z & X\prime W\\ Z\prime X & Z^{\prime }Z+I\lambda_{1} & Z\prime W\\ W\prime X & W\prime Z & WW+I\lambda_{2} \end{array}\right]\left[\begin{array}{c} \hat{b}\\ \hat{g}\\ \hat{c} \end{array}\right]=\left[\begin{array}{c} X\prime y\\ Z\prime y\\ W\prime y \end{array}\right]$$

In which:

$$\lambda_{1}=\frac{\sigma_{e}^{2}}{\sigma_{g}^{2}}\;e\;\lambda_{2}=\frac{\sigma_{e}^{2}}{\sigma_{gxe}^{2}}.$$

Estimators of variance components were estimated by REML via Expectation-Maximization algorithm as described below:

$${\hat{\sigma }}^{2}=\frac{\left[Y^{\prime }Y^{\prime }+{\hat{b}}^{\prime }X^{\prime }Y-{\hat{g}}^{\prime }Z^{\prime }Y-{\hat{c}}^{\prime }W^{\prime }Y\right]}{N-r(x)}$$

$${\hat{\sigma }}_{g}^{2}=\frac{\left[{\hat{g}}^{\prime }{\hat{g}}^{\prime }+{\hat{\sigma }}^{2}trC_{22}\right]}{q}$$

$${\hat{\sigma }}_{gxe}^{2}=\frac{\left[{\hat{c}}^{\prime }c+{\hat{\sigma }}^{2}trC_{33}\right]}{s}$$

where *C*_22_ e *C*_33_ is estimated by:

$$C^{-1}{\left[\begin{array}{ccc} C_{11} & C_{12} & C_{13}\\ C_{21} & C_{22} & C_{23}\\ C_{31} & C_{32} & C_{33} \end{array}\right]}^{-1}=\left[\begin{array}{ccc} C_{11} & C_{12} & C_{13}\\ C_{21} & C_{22} & C_{23}\\ C_{31} & C_{32} & C_{33} \end{array}\right]$$

in which: C is the coefficient matrix by mixed model equation; *tr* is the matrix trace; *r(x)* is the rank of the matrix X; *N* is the total of evaluated data points; *q* is the number of half-sib families; *s* is the number of GxE combinations.

The parameters heritability among half-sib families (*h*^2^), determination coefficient of the GxE interaction effects (*c*^*2*^); experimental coefficient of variation (CV_e_); genetic coefficient of variation (CV_g_), and CV_g_/CV_e_ ratio (CV_r_) were estimated by the equations described below:

$${\hat{h}}^{2}=\frac{\sigma_{g}^{2}}{{\sigma_{g}^{2}+\sigma }_{gxe}^{2}+\sigma_{e}^{2}}$$

$${\hat{c}}^{2}=\frac{\sigma_{gxe}^{2}}{{\sigma_{g}^{2}+\sigma }_{gxe}^{2}+\sigma_{e}^{2}}$$

$${CV}_{e}=\frac{\sqrt{{\hat{\sigma }}^{2}}}{\overset{-}{x}}$$

$${CV}_{g}=\frac{\sqrt{{\hat{\sigma }}_{g}^{2}}}{\overset{-}{x}}$$

$${CV}_{r}=\frac{{CV}_{g}}{{CV}_{e}}.$$

Adaptability and stability method proposed by Eberhart and Russell [8], is based on the simply linear regression analysis that measures the performance of each genotype over environment variations according to the equation below:

$$Y_{ij}=\beta_{0i}+\beta_{1i}I_{j}+\omega_{ij}$$

in which: Y_ij_ is the phenotypic value of the i^th^ half-sib family in the j^th^ environment; β_0i_ is the linear coefficient of the i^th^ half-sib family; β_1i_ is the regression coefficient that measure the i^th^ half-sib Family performance in the j^th^ environment; I_j_ is the environment index that is estimated by:

$$\left[I_{j}=\frac{\sum_{j}Y_{j}}{g}-\frac{\sum_{i}\sum_{j}Y_{ij}}{ga}\right]$$

In addition, *ω*_*ij*_ are the random error and it be decomposed as:

$$\omega_{ij}=\delta_{ij}+\overset{-}{\varepsilon_{ij}}$$

In which: δ_ij_ is the regression deviation, and ${\bar{\varepsilon }}_{\text{ij}}$ mean experimental error.

The estimative of mean square for adaptability and stability parameters based on Eberhart and Russell [8] are estimated according to:

$${\hat{\beta }}_{ij}=\frac{\sum_{j}Y_{ij}}{\sum_{j}I_{j}^{2}}$$

$${\hat{\sigma }}_{d_{i}}^{2}=\frac{{MSD}_{i}-{MS}_{r}}{r}$$

in which: MSD_i_ is the mean square deviation of the i^th^ half-sib family; *MS*_*r*_ is the residual mean square; e r é the number of replication.

The important hypothesis are: H_0_: β_1i_ = 1 *versus* H_1_: β_1i_ ≠ 1, and $H_{o}:\sigma_{di}^{2}=0$ *versus $H_{1}:\sigma_{di}^{2}>0$*. These hypotheses were evaluated by t and F test, respectively.

The method proposed by Cruz *et al*. [9], is based on the bi-segmented linear regression analysis according to the model below:

$$Y_{ij}=\beta_{0i}+\beta_{1i}I_{j}+\beta_{2i}T(I_{j})+\omega_{ij}$$

in which: Y_ij_ is the mean of the i^th^ genotypes in the j^th^ environments; β_0i_ is the linear coefficient of the i^th^ genotypes; β_1i_ is the linear performance of the i^th^ genotype for the unfavorable environment; β_1i_ + β_2i_ is the linear performance of the i^th^ genotypes for favorable environment; I_j_ is the environment index; T(I_j_) is equal zero if I_j_ < 0 or $\text{T}\left({\text{I}}_{\text{j}}\right)={\text{I}}_{\text{j}}-{\bar{\text{I}}}_{+}$ if I_j_ > 0, being ${\bar{\text{I}}}_{+}$ the mean of the positives environment indexes; ψ_ij_ are the random error for each component and it can be decomposed as: $\Psi_{\text{ij}}=\delta_{\text{ij}}+{\bar{\varepsilon }}_{\text{ij}}$, being δ_ij_ the regression deviation, and ${\bar{\varepsilon }}_{\text{ij}}$ the mean experimental error.

The estimators of minimum squares of the adaptability and stability parameters based on Cruz *et al*. [9] methods are calculated by the equations below:

$${\hat{\beta }}_{1i}=\frac{\sum_{j}Y_{ij}I_{j}-\sum_{j}Y_{ij}T(I_{j})}{\sum_{j}I_{j}^{2}-\sum_{j}T{\left(I_{j}\right)}^{2}}$$

$${\hat{\beta }}_{2i}=\frac{\sum_{j}I_{j}^{2}\sum_{j}Y_{ij}T(I_{j})-\sum_{j}T{\left(I_{j}\right)}^{2}\sum_{j}Y_{ij}I_{j}}{\sum_{j}T{\left(I_{j}\right)}^{2}[\sum_{j}I_{j}^{2}-\sum_{j}T{\left(I_{j}\right)}^{2}]}$$

$${\hat{\sigma }}_{d_{i}}^{2}=\frac{{QMD}_{i}-{QM}_{r}}{r}$$

The hypothesis are H_0_: β_1i_ = 1 *versus* H_1_: β_1i_ ≠ 1, H_0_: β_1i_ + β_2i_ = 1 *versus* H_1_: β_1i_ + β_2i_ ≠ 1, and ${\text{H}}_{\text{0}}{:\sigma }_{\text{di}}^{\text{2}}=0$ *versus ${\text{H}}_{\text{1}}{:\sigma }_{\text{di}}^{\text{2}}>0$*. The first two hypothesis were evaluated by t test and the last one was evaluated by F test, respectively.

Half-sib families were group into six groups based on Eberhart and Russell [8] and Cruz *et al*. [9] methods according to Table 1.

**Table 1:** Possibly groups based on Eberhart and Russell [8] (E&R) and Cruz *et al*. [9] (Cruz) methods.

Classes	Pratical classification	E&R	Cruz
1	General adaptability and low stability	β_1_ = 1 e $\sigma_{\text{di}}^{\text{2}}>0$	β_1_ = 1 e $\sigma_{\text{di}}^{\text{2}}>0$
2	Specific Adaptability for favorable environments and low stability	β_1_ > 1 e $\sigma_{\text{di}}^{\text{2}}>0$	β_1_ + β_2_ > 1 e $\sigma_{\text{di}}^{\text{2}}>0$
3	Specific Adaptability for unfavorable environments and low stability	β_1_ < 1 e $\sigma_{\text{di}}^{\text{2}}>0$	β_1_ < 1 e $\sigma_{\text{di}}^{\text{2}}>0$
4	General adaptability and high stability	β_1_ = 1 e $\sigma_{\text{di}}^{\text{2}}=0$	β_1_ = 1 e $\sigma_{\text{di}}^{\text{2}}=0$
5	Specific Adaptability for favorable environments and high stability	β_1_ > 1 e $\sigma_{\text{di}}^{\text{2}}=0$	β_1_ + β_2_ > 1 e $\sigma_{\text{di}}^{\text{2}}=0$
6	Specific Adaptability for unfavorable environments and high stability	β_1_ < 1 e $\sigma_{\text{di}}^{\text{2}}=0$	β_1_ < 1 e $\sigma_{\text{di}}^{\text{2}}=0$

The accuracy and genetic value based on Eberhart and Russell [8] and Cruz *et al*. [9] methods were estimated by the equation below proposed by Resende [13]:

$$Accuracy={\left(1-\frac{1}{F}\right)}^{2}$$

$$Genetic\;value=Halfsib\;familiy\;mean\;*\;(1-\frac{1}{F})$$

The harmonic mean values of the genotypic values (HMGV) to evaluate the stability based on the HMRPGV method was estimated according to the equation:

$${MHGV}_{i}=\frac{n}{\sum_{j=1}^{n}\frac{1}{{GV}_{ij}}}$$

In which n is the number of environment where the i^th^ genotype was evaluated, GV_ij_ is the genotypic value of the i^th^ genotype in the j^th^ environment, and it can be expressed as the mean proportion of the environments [14].

The relative performance of the genotypic values (RPGV) is used to evaluate adaptability by the HMRPGV method and it estimated by the equation below:

$${RPGV}_{i}=\frac{1}{n}\frac{\sum_{j=1}^{n}{GV}_{ij}}{M_{j}}$$

In which M_j_ is the trait mean (PROD or W100S) in the j^th^ environment.

A seleção conjunta considerando-se, simultaneamente, a PROD/W100S, a estabilidade e a adaptabilidade é dada pela estatística harmonic mean of the relative performance of genetic values (HMRPGV) [14]:

$${HMRPGV}_{i}=\frac{n}{\sum_{j=1}^{n}\frac{i}{{RPGV}_{i}}}$$

Therefore, genotypes with higher HMRPGV are those that have high adaptability and high stability simultaneously for the environments evaluated in this study for PROD and W100S.

For each method and each character, 20 families were selected for each method with general adaptability and high stability simultaneously. Subsequently, the coincidence index and predicted gain for the selection of each method were calculated. For each method, the 60 half-sib families of half-siblings that had high adaptability and predictability were selected simultaneously for the PROD during the first five years. From this selection, the prediction accuracy of PROD was calculated in the sixth production year, and it was also predicted the genotypic value for all of them. The prediction accuracy was estimated as being the squared Pearson correlation between the phenotypic value and the genotypic value. The genotypic value was predicted based on the betas estimated by Eberhart and Russell [8] and Cruz *et al*. [9] methods for each family.

All statistical analyses were performed using the software Genes [15] and Selegen [14].

## Results

### Estimative of genetic parameters

Analysis of variance (ANOVA) and mixed models were performed aiming to estimate genetic parameters for yield production (PROD) and weight of 100 seeds (W100S) evaluated during five years. It was verified that there is genetic variability among Jatropha half-sib families, and the genetic variance estimated by ANOVA was twice compared with the genetic variance estimated by mixed models (Table 2). It was also observed that the GxE interaction was significant, mainly for PROD. However, ANOVA was unable to estimate the GxE interaction accurate for W100S, due to the estimated value was out the parametric space (negative variance) (Table 2).

**Table 2:** Estimative of genetic parameters via analysis of variance (ANOVA) and mixed models for yield production (PROD) and weight of 100 seeds (W100S) evaluated in 180 Jatropha half-sib families during five years.

Parameters	ANOVA		Mixed models	
	PROD	W100S	PROD	W100S
Mean	993.65	65.79	994.14	65.77
${\hat{\sigma }}_{g}^{2}$	28894.19	14.32	14061.03	9.99
${\hat{\sigma }}_{p}^{2}$	*	*	28814.54	5.41
${\hat{\sigma }}_{ge}^{2}$	21324.51	-1.76	35409.10	1.44
${\hat{\sigma }}^{2}$	122592.63	19.50	93766.21	15.98
${\hat{\sigma }}_{f}^{2}$	172811.33	32.06	172050.88	32.84
${\hat{h}}^{2}$	0.64	0.88	0.31	0.66
${\hat{c}}^{2}$	0.12	0.00	0.25	0.04
CV_e_	35.24	5.75	35.24	5.75
CV_g_	17.11	6.71	21.50	5.36
CV_r_	0.49	0.86	0.68	0.81

${\hat{\sigma }}_{g}^{2}$: genetic variance; ${\hat{\sigma }}_{p}^{2}$: permanent effects variance; ${\hat{\sigma }}_{ge}^{2}$: genotypesXenvironment (GxE) interaction variance; ${\hat{\sigma }}^{2}$: residual variance; ${\hat{\sigma }}_{f}^{2}$: phenotypic variance; ${\hat{h}}^{2}$: heritability among half-sib families; ${\hat{c}}^{2}$: coefficient of determination of the GxE interaction effect; CV_e_: experimental coefficient of variation; CV_g_: genetic coefficient of variation; CV_r_: CV_g_/CV_e_ ratio;

*: effect not estimated in the ANOVA model.

The magnitude of heritability among half-sib families (${\hat{h}}^{2}$) estimated by ANOVA was higher than REML estimative (Table 2). Moreover, the magnitude of coefficient of determination for GxE interaction effects (${\hat{c}}^{2}$) was low for both traits according to de Resende [11]. Experimental coefficient of variation (CV_e_) was considered high for PROD (greater than 20%) and low for W100S (Table 2). CV_g_/CV_e_ ratio was lesser than one for both traits.

Because of the GxE interaction significance, ANOVA and mixed models were performed for each year separately (Table 3). It was verified that the genetic variance estimated by REML was greater than estimated by ANOVA in magnitude for both traits. However, the heritability was the same between methods for both traits and during the years.

**Table 3:** Estimative of genetic parameters via analysis of variance (ANOVA) and mixed models for yield production (PROD) and weight of 100 seeds (W100S) evaluated in the 180 half-sib families for each evaluated year.

Year	Mean	CV_e_	$\sigma_{g}^{2}$	h^2^
	**PROD—ANOVA**			
1	166.84	35.05	3139.57	0.65
2	465.72	34.84	7388.03	0.36
3	1242.81	28.64	88992.49	0.58
4	1157.24	40.70	23490.40	0.17
5	1935.63	25.03	128083.00	0.52
	**PROD—Mixed models**			
1	166.84	35.05	12558.02	0.65
2	465.72	34.84	29547.29	0.36
3	1242.81	28.64	355958.90	0.58
4	1157.24	40.70	93802.45	0.17
5	1935.63	25.03	512309.03	0.52
	**W100S—ANOVA**			
1	64.26	6.69	14.59	0.61
2	68.52	4.85	8.36	0.60
3	61.38	7.92	11.40	0.49
4	68.99	7.23	15.86	0.56
	**W100S—Mixed models**			
1	64.26	6.69	58.37	0.61
2	68.52	4.85	33.44	0.60
3	61.38	7.92	45.60	0.49
4	68.99	7.23	63.44	0.56

CV_e_: experimental coefficient of variation; **${\hat{\sigma }}_{g}^{2}$**: genetic variance; ${\hat{h}}^{2}$: heritability among half-sib families.

Genetic variance estimated by mixed models was four times greater than the genetic variance estimated by ANOVA for PROD and W100S (Table 3). The heritability for PROD ranged from 0.17 to 0.65 during the years, while the heritability for W100S ranged from 0.49 to 0.61 (Table 3). The CV_e_ was high (greater than 20%) for PROD in all evaluated years and low for W100S in all evaluated years (Table 3).

### Classification of the half-sib families based on adaptability and stability methods

Adaptability and stability analysis were performed using two methods widely used in the plant breeding aiming to identify superior genotypes for favorable and unfavorable environments: method proposed by Eberhart and Russell [8] and method proposed by Cruz *et al*. [9].

**Fig 1:**
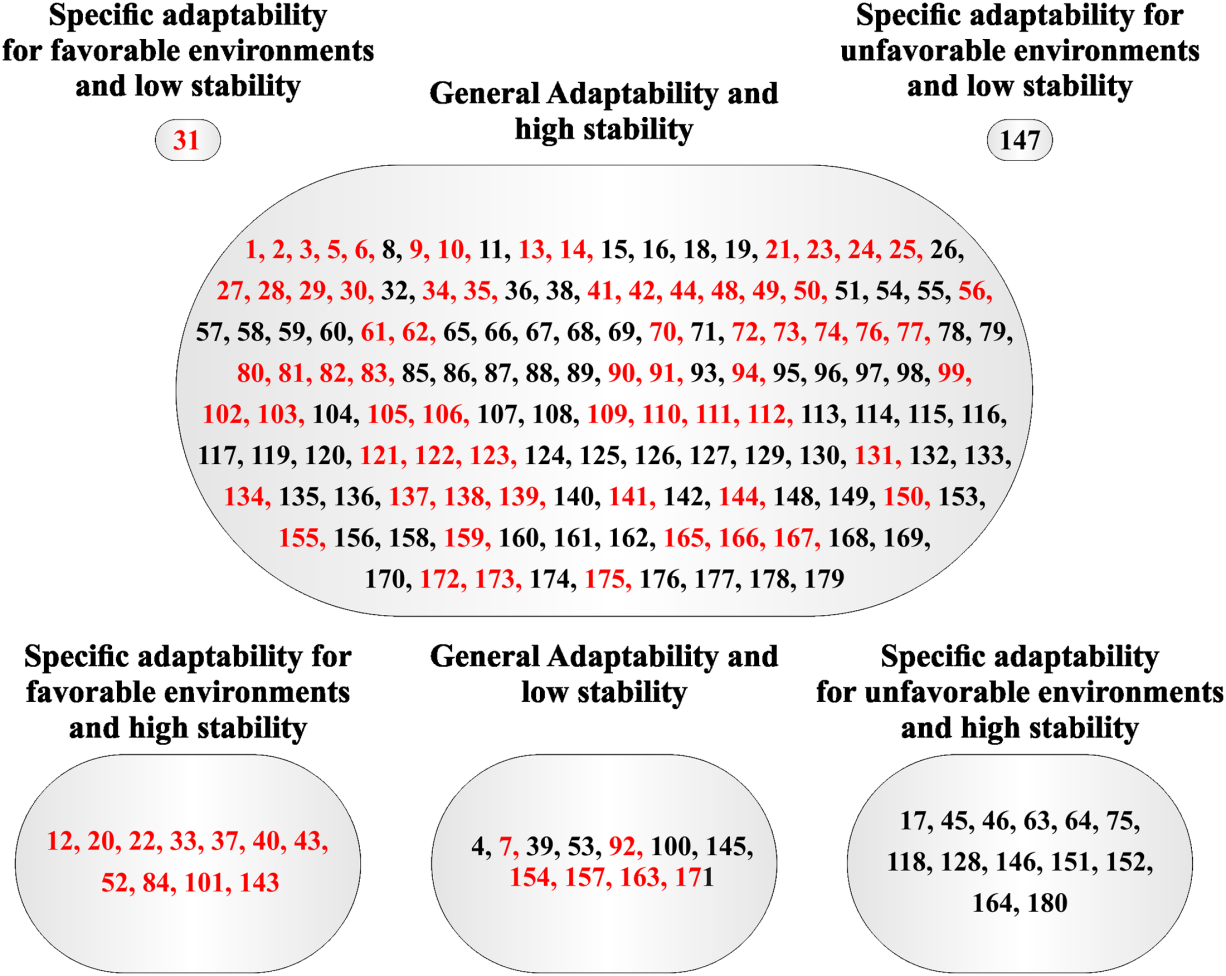
Classification of the 180 Jatropha half-sib families based on adaptability and stability parameters via Eberhart and Russell [8] method for yield production (PROD) evaluated during five years. Families highlighted with red color have mean superior with the overall mean.

**Fig 2:**
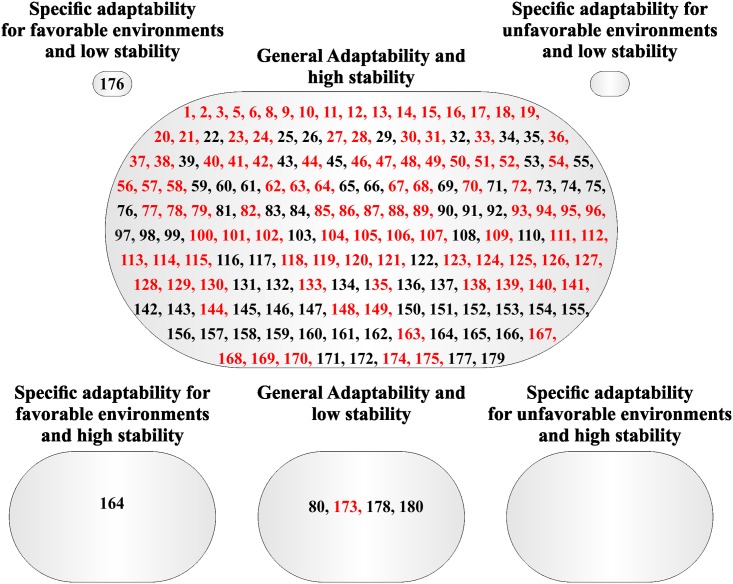
Classification of the 180 Jatropha half-sib families based on adaptability and stability parameters via Eberhart and Russell [8] method for weight of 100 seeds (W100S) evaluated during four years. Families highlighted with red color have mean superior with the overall mean.

Based on the method proposed by Eberhart and Russell [8] was verified a large group composed by families with general adaptability and high stability for PROD and W100S (Figs 1 and 2, respectively). In addition, three small groups were formed for PROD: specific adaptability for favorable environments and high stability (11 families), general adaptability and low stability (11 families), and specific adaptability for unfavorable environments and high stability (13 families) (Fig 1). On the other hand two groups were formed just by one family: specific adaptability for favorable environments and low stability, and specific adaptability for unfavorable environments and low stability (Fig 1).

General adaptability and low stability group was formed by four families for W100S (Fig 2), while specific adaptability for favorable environments and high stability, and specific adaptability for favorable environments and low stability groups were formed jus for one family. In addition, specific adaptability for favorable and unfavorable environments groups presented no families (Fig 2).

**Fig 3:**
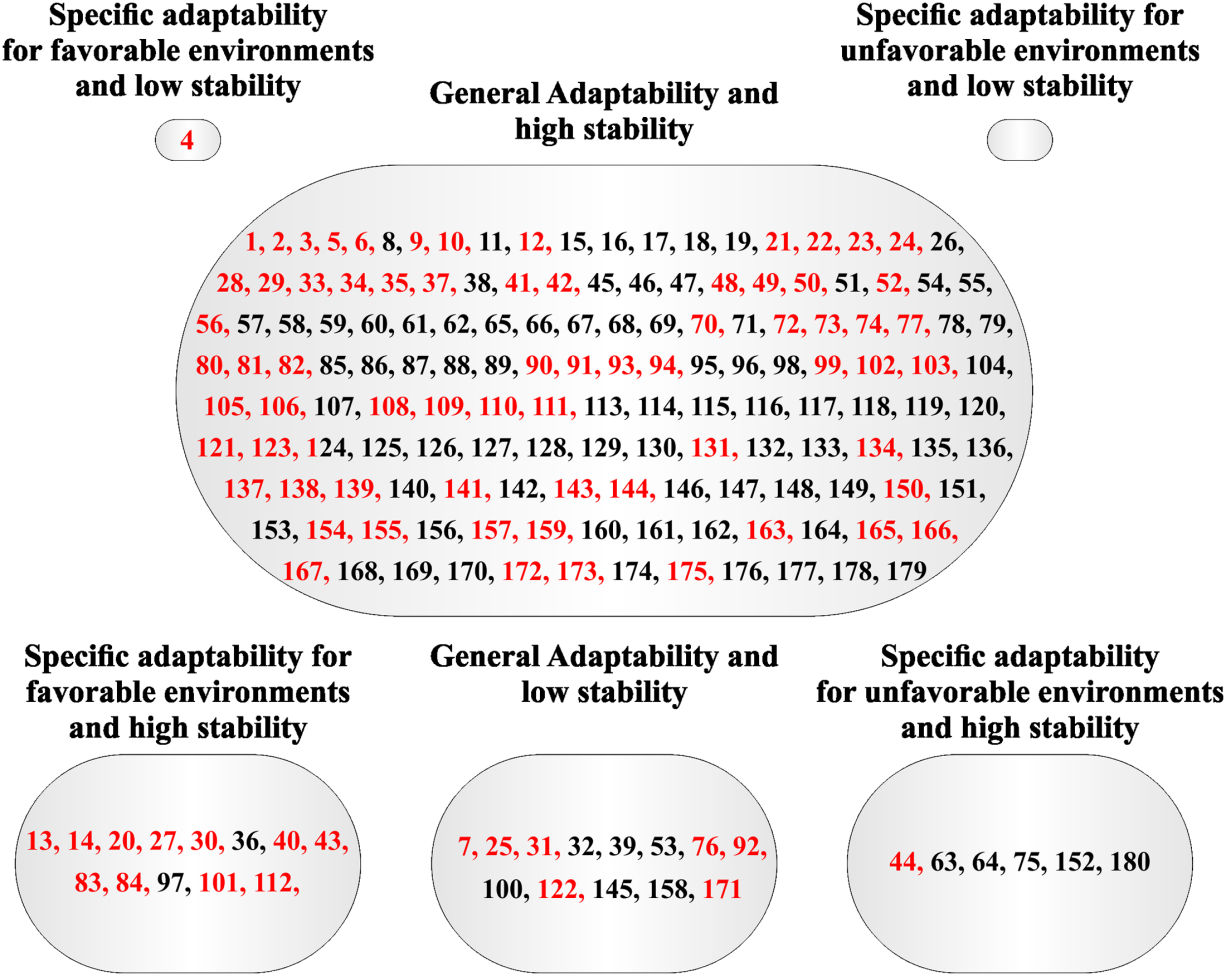
Classification of the 180 Jatropha half-sib families based on adaptability and stability parameters via Cruz *et al*. [9] method for yield production (PROD) evaluated during five years. Families highlighted with red color have mean superior with the overall mean.

**Fig 4:**
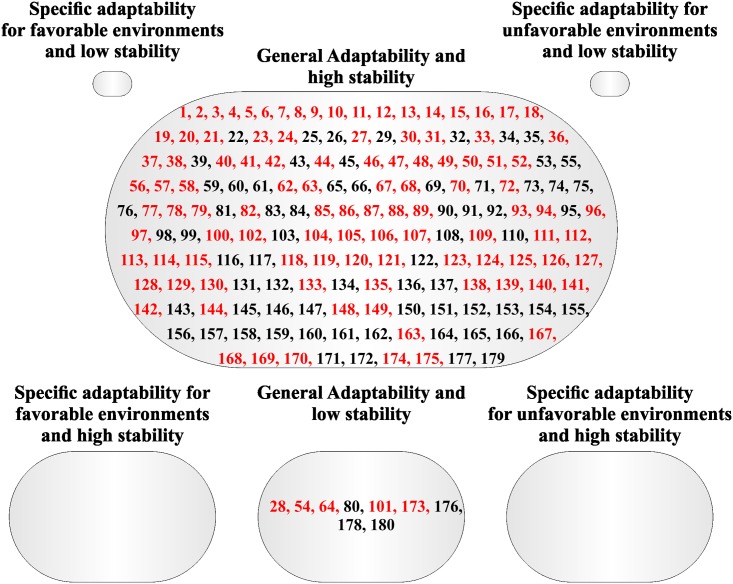
Classification of the 180 Jatropha half-sib families based on adaptability and stability parameters via Cruz *et al*. [9] method for weight of 100 seeds (W100S) evaluated during four years. Families highlighted with red color have mean superior with the overall mean.

A large group with general adaptability and high stability was also formed by the method proposed by Cruz *et al*. [9] for PROD and W100S (Figs 3 and 4). In addition, three small groups were formed for PROD: specific adaptability for favorable environments and high stability (13 families), general adaptability and low stability (13 families), and specific adaptability for unfavorable environments and high stability (six families) (Fig 3). One family formed the group specific adaptability for favorable environments and low stability for PROD (Fig 3). Moreover the group general adaptability and low stability was formed by nine families for W100S (Fig 4), whereas the others groups had no families for W100S.

Families highlighted with red in the Figs 1, 2, 3 and 4 had mean greater than the overall mean for PROD and W100S. It was observed that most all families with mean superior than the overall mean belonged to the group with general adaptability and high stability for both methodologies. For PROD, it was possible to verify families with mean superior than the overall mean in the groups with general adaptability and low stability, and specific adaptability for favorable environment and high (Figs 1 and 3).

### Family selection and estimation of selection gain via adaptability and stability methods

Eberhart and Russell [8], Cruz *et al*. [9], and HMRPGV were performed aiming to select the 20 superior Jatropha half-sib families with general adaptability and high stability. The prediction accuracy was higher for Cruz *et al*. [9] and Eberhart and Russell [8] methods compared with HMRPGV for PROD and W100S (Tables 4 and 5, respectively). The betas estimative calculated by Eberhart and Russell [8] and Cruz *et al*. [9] methods for the 20 superior half-sib families were presented in the S1 and S2 Tables.

**Table 4:** Selected Jatropha half-sib families via adaptability and stability methods for yield production (PROD), and their genetic values and individual predicted accuracy.

Eberhart and Russell [8]			Cruz *et al*. [9]			HMRPGV		
Genotypes	Genetic Value	Accuracy	Genotypes	Genetic Value	Accuracy	Genotypes	Genetic Value	Accuracy
31	994.75	0.80	31	1166.18	0.87	31	1164.31	0.56
22	720.55	0.69	22	979.72	0.81	33	1166.32	0.56
61	758.73	0.74	61	602.69	0.66	61	1117.79	0.56
1	786.29	0.76	1	900.15	0.81	12	1140.6	0.56
92	839.24	0.79	92	969.91	0.85	101	1103.22	0.56
20	679.39	0.72	7	1066.99	0.92	22	1152.33	0.56
163	896.56	0.83	122	869.91	0.83	81	1094.29	0.56
122	743.84	0.77	175	715.19	0.75	139	1093.50	0.56
175	548.78	0.66	6	525.33	0.65	94	1076.20	0.56
6	572.19	0.68	171	1117.72	0.95	92	1106.31	0.56
76	627.43	0.72	111	236.40	0.44	173	1064.00	0.56
40	738.41	0.78	4	928.34	0.88	1	1110.38	0.56
30	720.44	0.78	76	817.15	0.83	76	1058.64	0.56
154	734.76	0.79	40	555.07	0.68	172	1048.67	0.56
159	696.51	0.77	30	684.00	0.76	83	1064.71	0.56
155	542.04	0.68	159	761.36	0.80	82	1075.26	0.56
62	555.87	0.70	62	741.57	0.81	163	1085.61	0.56
25	660.83	0.77	80	235.74	0.46	84	1075.43	0.56
9	651.60	0.77	25	772.63	0.83	74	1047.01	0.56
157	858.11	0.89	5	573.58	0.72	143	1105.13	0.56
Accuracy		0.85		0.85				0.56

HMRPGV—Harmonic mean of the relative performance of genetic values.

**Table 5:** Selected Jatropha half-sib families via adaptability and stability methods for weight of 100 seeds (W100S), and their genetic values and individual predicted accuracy.

Eberhart and Russell [8]			Cruz *et al*. [9]			HMRPGV		
Genotypes	Genetic value	Accuracy	Genotypes	Genetic value	Accuracy	Genotypes	Genetic value	Accuracy
28	38.66	0.73	28	53.76	0.86	20	70.67	0.81
173	65.52	0.96	173	68.62	0.98	28	70.33	0.81
63	36.73	0.72	63	46.44	0.81	173	69.86	0.81
1	11.86	0.42	10	2.54	0.19	79	69.55	0.81
82	1.47	0.15	13	20.28	0.54	17	69.39	0.81
101	35.67	0.73	1	35.25	0.72	19	69.29	0.81
54	40.13	0.77	82	27.42	0.63	9	69.22	0.81
27	1.21	0.13	101	50.89	0.87	93	69.17	0.81
104	2.87	0.21	54	53.60	0.89	63	69.16	0.81
30	7.13	0.33	27	31.41	0.68	37	69.09	0.81
64	38.54	0.76	104	23.14	0.59	21	69.09	0.81
7	14.92	0.47	64	52.91	0.89	58	68.98	0.81
15	1.45	0.15	7	29.59	0.66	40	68.91	0.81
57	39.04	0.77	175	19.22	0.53	169	68.88	0.81
11	3.63	0.23	109	27.89	0.65	1	68.86	0.81
147	40.99	0.79	57	38.89	0.77	115	68.79	0.81
80	43.36	0.82	170	30.48	0.68	10	68.66	0.81
143	18.19	0.53	74	11.61	0.42	56	68.65	0.81
76	29.34	0.68	61	19.89	0.55	174	68.57	0.81
95	41.52	0.81	80	53.88	0.91	31	68.43	0.81
Accuracy		0.92		0.92				0.81

HMRPGV—Harmonic mean of the relative performance of genetic values.

The coincidence index among the adaptability and stability methods was calculated and it was verified that there was a high coincidence among the selected Jatropha half-sib families between Eberhart and Russell [8] and Cruz *et al*. [9] methods for PROD and W100S (Table 6). On the other hand, the coincidence between these methods and HMRPGV was low for PROD and W100S.

**Table 6:** Coincidence index among adaptability and stability methods based on the 20 superior Jatropha half-sib families for yield production (PROD–superior diagonal) and weight of 100 seeds (W100S –inferior diagonal).

**Methods**	**E&R**	**Cruz**	**HMRPGV**
**E&R**	-	0.70	0.35
**Cruz**	0.55	-	0.30
**HMRPGV**	0.20	0.25	-

E&R–adaptability and stability method proposed by Eberhart and Russell [8]; Cruz–adaptability and stability method proposed by Cruz *et al*. [9]; HMRPGV—Harmonic mean of the relative performance of genetic values.

The selection gain estimated by the HMRPGV was greater than the selection gain estimated by the other methods for PROD e W100S (Table 7). The selection gain in percentage for PROD ranged from (HMRPGV) to 9 (Eberhart and Russell [8] and Cruz *et al*. [9] methods) times greater than the selection gain for W100S.

**Table 7:** Estimative of selection gain based on the 20 superior Jatropha half-sib families via adaptability and stability methods for yield production (PROD) and weight of 100 seeds (W100S).

Methods	${\overset{-}{X}}_{p}$	${\overset{-}{X}}_{S}$	SG	SG (%)
	**PROD**			
**E&R**	993.65	1252.22	165.48	16.65
**Cruz**	993.65	1253.62	166.38	16.74
**HMRPGV**	994.01	1197.49	203.48	20.47
	**W100S**			
**E&R**	65.79	67.21	1.25	1.89
**Cruz**	65.79	67.66	1.65	2.50
**HMRPGV**	65.77	69.18	3.41	5.18

E&R–adaptability and stability method proposed by Eberhart and Russell [8]; Cruz–adaptability and stability method proposed by Cruz *et al*. [9]; HMRPGV–Harmonic mean of the relative performance of genetic values; ${\overset{-}{X}}_{p}–$ population overall mean; ${\overset{-}{X}}_{S}–$ selected population mean; SG–selection gain; SG (%)–selection gain in percentage.

The prediction accuracy for the sixth production year based on the 60 superior Jatropha half-sib families selected after the fifth production year estimated by Eberhart and Russell [8] and Cruz *et al*. [9] methods was 0.45 for both methods. The beta values of the Jatropha half-sib families evaluated in the sixth production year and their genotypic values for PROD are shown in S3 Table.

## Discussion

### Estimative of genetic parameters

Differences among the estimative of genetic parameters and variance components found in this study was also reported previously in researches comparing ANOVA and REML [16], and it can be happened due to the unbalanced data because there were missing plants in the plots. Moreover, REML presented lower estimative because it considers basically the random effects in the statistic model associated with the phenotypic values, which the data are adjusted for the fixed effects and the unequal number of plants per plot based on mixed models [17, 18].

GxE interaction is one of the most challenge in the plant breeding because it makes the genotypes recommendation for many environments more difficult [7]. Indeed, Jatropha is still considered a undomesticated crop in Brazil, and consequently there is a few researches evaluating the GxE interaction [19, 20]. As the environments evaluated in this study were production years, the difference among Jatropha half-sib families in the environments can be assigned by climate factors such as precipitation, temperature and humidity that interfered in the genotype performance during the years, and consequently these factors make the GxE interaction signiticative. Therefore, adaptability and stability analysis is a useful tool to help breeders to select superior Jatropha half-sib more accurate for W100S and PROD.

Heritability ${\hat{h}}^{2}$ estimated by ANOVA was higher than ${\hat{h}}^{2}$ estimated by REML, due to ANOVA overestimated ${\hat{\sigma }}_{g}^{2}$. Because that REM is more recomended to analyze experiments with unbalanced data. In addition, the magnitude of the h^2^ estimated by REML were similar with h^2^ reported previously in the literature for W100S and PROD [21–24]. High CV_e_ for PROD and low for W100S have also reported in the literature previously [20, 25].

There was a drastic reduction of the number of observation for estimating genetic parameters for each year in separate analysis, and it made that ${\hat{\sigma }}_{g}^{2}$ was higher when it was estimated by REML due to this method tends overestimate the ${\hat{\sigma }}_{g}^{2}$ in experiments with reduced number of data points.[26]. Furthermore, how the number of missing data was reduced when each year was evaluated separately, the ${\hat{h}}^{2}$ was the same for both methodologies for all production years. According to de Farias Neto and de Resende [18] REML and ANOVA have to take the same results or with a slightly difference when the experiment is balanced, and therefore, for this case ANOVA is efficient.

### Family selection and selection gain estimates via adaptability and stability methods

The identification of genotypes adapted for favorable and unfavorable environments is important in the Jatropha breeding and it will made possible to expand the cultivation of this important crop for other Brazilian regions. Genotypes identified having specific adaptability for favorable environments, high PROD average, and high stability might be used for farmers with high technological level, due to these genotypes are able to improve their performance with the environment conditions improvement.

On the other hand, genotypes with specific adaptability for unfavorable environments, high PROD average, and high stability may be planted by farmers with low technological level, because these genotypes have high rusticity and consequently they will maintain the yield production even they will be submitted for adverse conditions.

In addition, genotypes with general adaptability, high PROD average, and high stability can be also cultivated in all environments, however their performance will be lower compared with genotypes having specific adaptability for favorable environments with the environment conditions improvement, and they will be a more seasonal performance compared with genotypes that have specific adaptability for unfavorable environments.

Interestingly, the Jatropha half-sib Family classification for W100S based on adaptability and stability methods is important because this trait is highly positive related with PROD [25], and it also has higher heritability and it is easy to measure. Therefore, genotypes that have high stability and high average for this trait can be used in the Jatropha breeding aiming to make indirect selection for PROD in favorable and unfavorable environments.

Eberhart and Russell [8], Cruz *et al*. [9] and HMRPGV selected different half-sib families for PROD and W100S. However, the coincidence index between Eberhart and Russell [8] and Cruz *et al*. [9] were high. These methods based on linear regression are widely used to identify and to select genotypes with high adaptability and stability. However it is needed to consider that the environmental mean is composed by the environments effects plus the GxE interaction, and it can lead on any mistakes about the real causes of the GxE interaction [27]. Besides that, according to Duarte [28] genotypes behavior may not be a linear relationship with the environment.

Genotypic value predicted by HMRPVG were higher than the genotypic values predicted by other methods. These results were expected because the genotypic value predicted by HMRPVG are expressed as the proportion of the overall mean for each environment, which penalizing the genotypes with low stability. This methodology allows maximizing the selection gain compared with other adaptability and stability methods for PROD and W100S. Besides that, the genotype recommendation is ease in this method because this method ranked the genotypes based on the HMRPGV, and this value is calculated based on the genotype mean, adaptability and stability. Therefore, genotypes with high HMRPGV value have high mean, adaptability, and stability.

### Future applications

Jatropha is a perennial specie that has been widely used to oil production, and it can also be reported that it is possible to obtain gain with selection for this crop based on the genetic information. Therefore, the use of currently techniques performed in the Jatropha breeding, plus statistical methodologies more robust such as the HMRPGV, and the use of molecular markers such as Single Nucleotide Polymorphism (SNP) can be the key to improve the selection accuracy, to reduce the cycle time, and to decrease the cost per cycle time.

The use of SNPs should be exploited and applied in the future researches aiming to improve the prediction accuracy. Recent developments of the next-generation sequencing platforms has allowed researchers genotype populations with a large number of individuals quickly and with low cost [29].

Therefore, the use of molecular markers exploiting the GxE interaction has emerged as a useful strategy to select superior genotypes, especially in forest species which the cycle time is too large [30] and evaluations should be performed when the genotypes stabilized their yield production, that happens around 11 years old in Jatropha. Based on the theoretical and practical studies as discussed in this research, the introduction of genome-wide selection models performed with the GxE interaction will may increase the efficiency of the Jatropha breeding reducing the cycle time, and/or omitting the progeny test phase. [31].

## Conclusion

There are genetic variability among Jatropha half-sib Family;

GxE interaction was statistically significant for both traits and the adaptability and stability methods were able to split favorable and unfavorable environments;

HMRPGV is efficient to maximize the genetic gain compared with other methods, making it a suitable method to select superior genotypes in long cycle species;

The half-sib families 20, 40, 43, and 101 have high-yield production, specific adaptability for favorable environments and high stability;

The half-sib family 44 has high-yield production, specific adaptabil8ity for unfavorable environments and high stability;

Cruz *et al*. [9] and [Eberhart and Russell [8]] were able to predict the sixth production year based on the selected half = sib Family with a moderate accuracy.

## Supporting information

S1 Table β_0_ and β_1_ values of the Jatropha full-sib families selected via adaptability and stability method for yield production (PROD). (DOCX)

S2 Table β_0_ and β_1_ values of the Jatropha full-sib families selected via adaptability and stability method for weight of 100 seeds (W100S). (DOCX)

S3 Table Betas values of the Jatropha half-sib families evaluated in the sixth production year and their genotypic values (GV) for PROD. (DOCX)

S1 Dataset Dataset used to run all statistical analysis. (XLSX)
