# Supplementary material for: Jatropha half-sib family selection with high adaptability and genotypic stability
Source: PLoS One. 2018 Jul 12;13(7):e0199880. doi: 10.1371/journal.pone.0199880 (PMC6042709; doi:10.1371/journal.pone.0199880)
Supplement: S1 Table — (DOCX) [file pone.0199880.s001.docx]

**S1 Table.** β_0_ and β_1_ values of the Jatropha full-sib families selected via adaptability and stability method for yield production (PROD).

| [**Eberhart & Russell (1966)**](#_ENREF_13) | | | [**Cruz et al. (1989)**](#_ENREF_5) | | | |
| --- | --- | --- | --- | --- | --- | --- |
| **Genotypes** | **Β_0_** | **Β_1_** | **Genotypes** | **Β_0_** | **Β_1_** | **Β_2_** |
| 31 | 1537.80 | **1.49** | 31 | 1537.80 | **1.53** | -0.21 |
| 22 | 1499.54 | **1.72** | 22 | 1499.54 | **1.71** | 0.03 |
| 61 | 1389.18 | 1.12 | 61 | 1462.05 | 1.27 | -0.80 |
| 1 | 1365.51 | 1.32 | 1 | 1389.18 | **1.42** | -0.50 |
| 92 | 1352.51 | 1.33 | 92 | 1352.51 | **1.41** | -0.42 |
| 20 | 1316.25 | **1.59** | 7 | 1348.73 | 1.32 | **-0.85** |
| 163 | 1286.36 | 1.14 | 122 | 1342.64 | 1.28 | -0.43 |
| 122 | 1266.13 | 1.20 | 175 | 1316.25 | 1.36 | -0.38 |
| 175 | 1255.71 | 1.29 | 6 | 1229.59 | 1.06 | 0.66 |
| 6 | 1229.59 | 1.19 | 171 | 1227.43 | 1.11 | 0.46 |
| 76 | 1200.23 | 1.02 | 111 | 1227.27 | 1.26 | 0.16 |
| 40 | 1198.82 | **1.51** | 4 | 1219.60 | 0.91 | **0.94** |
| 30 | 1188.03 | 1.24 | 76 | 1200.23 | 1.00 | 0.06 |
| 154 | 1182.96 | 0.97 | 40 | 1198.82 | 1.34 | **0.92** |
| 159 | 1175.91 | 1.01 | 30 | 1191.41 | 1.09 | 0.78 |
| 155 | 1171.96 | 0.99 | 159 | 1188.03 | 1.13 | -0.59 |
| 62 | 1129.96 | 1.08 | 62 | 1129.96 | 1.11 | -0.13 |
| 25 | 1119.65 | 1.00 | 80 | 1129.67 | 1.30 | 0.21 |
| 9 | 1089.89 | 1.20 | 25 | 1119.66 | 1.08 | -0.42 |
| 157 | 1088.48 | 0.77 | 5 | 1118.94 | 0.95 | 0.23 |

Bold estimative are statistically different than 1 by the t Test of 5% probability.
