# Supplementary material for: Jatropha half-sib family selection with high adaptability and genotypic stability
Source: PLoS One. 2018 Jul 12;13(7):e0199880. doi: 10.1371/journal.pone.0199880 (PMC6042709; doi:10.1371/journal.pone.0199880)
Supplement: S2 Table — (DOCX) [file pone.0199880.s002.docx]

**S2 Table.** β_0_ and β_1_ values of the Jatropha full-sib families selected via adaptability and stability method for weight of 100 seeds (W100S).

| [**Eberhart & Russell (1966)**](#_ENREF_13) | | | [**Cruz et al. (1989)**](#_ENREF_5) | | | |
| --- | --- | --- | --- | --- | --- | --- |
| **Genotypes** | **Β_0_** | **Β_1_** | **Genotypes** | **Β_0_** | **Β_1_** | **Β_2_** |
| 28 | 72.40 | 0.93 | 28 | 72.40 | 0.91 | 6.05 |
| 173 | 71.72 | 0.75 | 173 | 71.72 | 0.75 | 0.56 |
| 63 | 70.71 | 1.31 | 63 | 70.71 | 1.28 | 10.60 |
| 1 | 68.77 | 1.70 | 10 | 69.97 | 1.01 | 8.70 |
| 82 | 68.37 | 0.76 | 13 | 69.20 | 1.04 | -0.69 |
| 101 | 67.69 | 1.39 | 1 | 68.77 | 1.69 | 5.73 |
| 54 | 67.51 | 1.29 | 82 | 68.37 | 0.78 | -5.80 |
| 27 | 67.48 | 1.00 | 101 | 67.69 | 1.40 | -4.22 |
| 104 | 67.46 | 1.31 | 54 | 67.51 | 1.29 | -2.67 |
| 30 | 67.44 | 0.68 | 27 | 67.48 | 0.99 | 3.86 |
| 64 | 67.33 | 1.02 | 104 | 67.46 | 1.29 | 7.14 |
| 7 | 67.31 | 1.60 | 64 | 67.33 | 1.02 | 0.85 |
| 15 | 67.10 | 1.16 | 7 | 67.31 | 1.63 | -8.41 |
| 57 | 66.35 | 1.28 | 175 | 67.19 | 0.15 | 2.16 |
| 11 | 66.34 | 1.29 | 109 | 66.76 | 1.33 | 4.73 |
| 147 | 65.32 | 0.15 | 57 | 66.35 | 1.24 | 14.83 |
| 80 | 64.47 | 1.28 | 170 | 66.04 | 1.41 | -0.84 |
| 143 | 64.06 | 1.03 | 74 | 65.35 | 1.23 | 2.92 |
| 76 | 63.61 | 1.50 | 61 | 64.76 | 1.25 | -1.55 |
| 95 | 62.76 | 0.39 | 80 | 64.47 | 1.28 | 1.31 |
