# Supplementary material for: Jatropha half-sib family selection with high adaptability and genotypic stability
Source: PLoS One. 2018 Jul 12;13(7):e0199880. doi: 10.1371/journal.pone.0199880 (PMC6042709; doi:10.1371/journal.pone.0199880)
Supplement: S3 Table — (DOCX) [file pone.0199880.s003.docx]

**S3 Table**. Betas values of the Jatropha half-sib families evaluated in the sixth production year and their genotypic values (GV) for PROD.

|  |  | [**Eberhart & Russell (1966)**](#_ENREF_13) | | | [**Cruz et al. (1989)**](#_ENREF_5) | | | |
| --- | --- | --- | --- | --- | --- | --- | --- | --- |
| **Genotypes** | **PROD** | **Β_0_** | **Β_1_** | **GV** | **Β_0_** | **Β_1_** | **Β_2_** | **GV** |
| 101 | 3033.0 | 1365.51 | 1.32 | 1373.46 | 1365.51 | 1.42 | -0.50 | 1374.02 |
| 104 | 2532.1 | 1203.71 | 1.09 | 1210.23 | 1203.71 | 0.91 | 0.94 | 1209.17 |
| 105 | 2152.6 | 1118.94 | 0.99 | 1124.90 | 1118.94 | 0.95 | 0.23 | 1124.64 |
| 106 | 2879.8 | 1229.59 | 1.19 | 1236.71 | 1229.59 | 1.06 | 0.66 | 1235.97 |
| 107 | 2496.7 | 1268.08 | 1.16 | 1275.04 | 1268.08 | 1.32 | -0.85 | 1276.00 |
| 113 | 3382.2 | 1462.05 | 1.44 | 1470.69 | 1462.05 | 1.55 | -0.59 | 1471.36 |
| 114 | 2300.4 | 1088.02 | 1.20 | 1095.24 | 1088.02 | 1.09 | 0.59 | 1094.58 |
| 116 | 2130.8 | 794.92 | 0.94 | 800.58 | 794.92 | 0.73 | 1.15 | 799.28 |
| 122 | 2720.3 | 1316.25 | 1.59 | 1325.81 | 1316.25 | 1.42 | 0.94 | 1324.75 |
| 124 | 2876.2 | 1499.54 | 1.72 | 1509.86 | 1499.54 | 1.71 | 0.03 | 1509.82 |
| 126 | 3200.4 | 1191.41 | 1.20 | 1198.62 | 1191.41 | 1.15 | 0.25 | 1198.34 |
| 127 | 2170.0 | 1119.65 | 1.00 | 1125.64 | 1119.65 | 1.08 | -0.42 | 1126.12 |
| 131 | 2406.8 | 1157.13 | 1.18 | 1164.21 | 1157.13 | 1.19 | -0.07 | 1164.29 |
| 132 | 2459.6 | 1188.03 | 1.24 | 1195.47 | 1188.03 | 1.09 | 0.78 | 1194.60 |
| 133 | 2700.8 | 1537.80 | 1.49 | 1546.72 | 1537.80 | 1.53 | -0.21 | 1546.96 |
| 136 | 2878.6 | 1544.24 | 1.51 | 1553.30 | 1544.24 | 1.66 | -0.78 | 1554.18 |
| 137 | 2430.2 | 1163.68 | 1.11 | 1170.33 | 1163.68 | 1.04 | 0.38 | 1169.91 |
| 140 | 2570.0 | 1227.27 | 1.39 | 1235.63 | 1227.27 | 1.32 | 0.39 | 1235.19 |
| 151 | 1226.2 | 502.76 | 0.30 | 504.57 | 502.76 | 0.38 | -0.39 | 505.01 |
| 154 | 2335.0 | 1013.67 | 1.20 | 1020.84 | 1013.67 | 1.15 | 0.23 | 1020.59 |
| 155 | 2226.7 | 1034.02 | 1.03 | 1040.18 | 1034.02 | 1.16 | -0.68 | 1040.95 |
| 161 | 2306.8 | 1094.94 | 1.04 | 1101.15 | 1094.94 | 1.00 | 0.18 | 1100.95 |
| 164 | 2548.2 | 668.06 | 0.83 | 673.02 | 668.06 | 0.73 | 0.51 | 672.44 |
| 167 | 1389.6 | 1389.18 | 1.12 | 1395.89 | 1389.18 | 1.27 | -0.80 | 1396.79 |
| 168 | 2611.0 | 1129.96 | 1.08 | 1136.46 | 1129.96 | 1.11 | -0.13 | 1136.61 |
| 169 | 606.0 | 562.89 | 0.38 | 565.16 | 562.89 | 0.54 | -0.87 | 566.15 |
| 170 | 220.0 | 198.12 | 0.02 | 198.24 | 198.12 | 0.06 | -0.21 | 198.49 |
| 180 | 2130.6 | 1022.35 | 0.80 | 1027.17 | 1022.35 | 0.93 | -0.68 | 1027.93 |
| 181 | 3115.8 | 1296.86 | 1.29 | 1304.60 | 1296.86 | 1.40 | -0.58 | 1305.25 |
| 182 | 2256.4 | 1163.07 | 1.15 | 1169.95 | 1163.07 | 1.05 | 0.51 | 1169.37 |
| 183 | 522.9 | 439.54 | 0.14 | 440.40 | 439.54 | 0.36 | -1.17 | 441.72 |
| 185 | 2892.0 | 1200.23 | 1.02 | 1206.33 | 1200.23 | 1.00 | 0.06 | 1206.26 |
| 188 | 1288.0 | 742.85 | 0.82 | 747.80 | 742.85 | 0.79 | 0.18 | 747.60 |
| 190 | 3218.6 | 1314.10 | 1.19 | 1321.26 | 1314.10 | 1.19 | 0.01 | 1321.25 |
| 191 | 3066.6 | 1253.31 | 1.18 | 1260.37 | 1253.31 | 1.21 | -0.16 | 1260.55 |
| 192 | 2977.6 | 1219.60 | 1.26 | 1227.13 | 1219.60 | 1.11 | 0.77 | 1226.27 |
| 193 | 3179.2 | 1253.86 | 1.40 | 1262.28 | 1253.86 | 1.25 | 0.84 | 1261.34 |
| 199 | 3020.0 | 1073.09 | 1.07 | 1079.49 | 1073.09 | 1.09 | -0.13 | 1079.64 |
| 201 | 3203.3 | 1352.51 | 1.33 | 1360.47 | 1352.51 | 1.41 | -0.42 | 1360.94 |
| 203 | 2536.1 | 1256.30 | 1.09 | 1262.86 | 1256.30 | 1.18 | -0.46 | 1263.38 |
| 210 | 3270.8 | 1342.64 | 1.44 | 1351.26 | 1342.64 | 1.33 | 0.56 | 1350.64 |
| 211 | 2932.6 | 1269.74 | 1.23 | 1277.12 | 1269.74 | 1.39 | -0.82 | 1278.05 |
| 212 | 2517.3 | 1153.18 | 1.14 | 1160.03 | 1153.18 | 1.25 | -0.59 | 1160.69 |
| 215 | 2422.0 | 1070.84 | 0.98 | 1076.72 | 1070.84 | 1.03 | -0.27 | 1077.02 |
| 216 | 3043.4 | 1169.71 | 1.20 | 1176.88 | 119.71 | 1.31 | -0.59 | 1177.55 |
| 220 | 2911.6 | 1064.75 | 1.08 | 1071.22 | 1064.75 | 1.16 | -0.43 | 1071.70 |
| 221 | 2878.2 | 1215.91 | 1.29 | 1223.66 | 1215.91 | 1.26 | 0.16 | 1223.48 |
| 232 | 3261.1 | 1266.13 | 1.20 | 1273.35 | 1266.13 | 1.28 | -0.43 | 1273.83 |
| 233 | 2180.6 | 1074.35 | 0.83 | 1079.33 | 1074.35 | 0.95 | -0.65 | 1080.06 |
| 234 | 2352.6 | 981.68 | 0.79 | 986.41 | 981.68 | 0.94 | -0.80 | 987.31 |
| 247 | 2162.8 | 1034.02 | 0.94 | 1039.68 | 1034.02 | 1.14 | -1.04 | 1040.85 |
| 252 | 2624.0 | 1014.22 | 0.90 | 1019.64 | 1014.22 | 1.03 | -0.68 | 1020.40 |
| 253 | 3494.4 | 1134.82 | 1.06 | 1141.20 | 1134.82 | 1.22 | -0.84 | 1142.15 |
| 254 | 3283.8 | 1311.58 | 1.30 | 1319.36 | 1311.58 | 1.35 | -0.28 | 1319.68 |
| 255 | 2564.0 | 967.95 | 0.78 | 972.63 | 967.95 | 0.86 | -0.44 | 973.13 |
| 258 | 3037.0 | 1348.73 | 1.47 | 1357.54 | 1348.73 | 1.55 | -0.41 | 1358.00 |
| 260 | 2995.0 | 1079.28 | 1.06 | 1085.64 | 1079.28 | 1.09 | -0.14 | 1085.80 |
| 265 | 3322.2 | 726.07 | 0.85 | 731.17 | 726.07 | 0.72 | 0.68 | 730.41 |
| 266 | 2937.8 | 1045.16 | 0.93 | 1050.74 | 1045.16 | 1.14 | -1.10 | 1051.99 |
| 270 | 3360.4 | 1182.96 | 0.97 | 1188.78 | 1182.96 | 1.21 | -1.27 | 1190.21 |
| 271 | 3279.6 | 1171.96 | 0.99 | 1177.90 | 1171.96 | 1.15 | -0.87 | 1178.88 |
| 272 | 3122.6 | 856.15 | 0.77 | 860.79 | 856.15 | 0.86 | -0.47 | 861.31 |
| 274 | 3108.2 | 1088.48 | 0.77 | 1093.10 | 1088.48 | 1.09 | -1.71 | 1095.03 |
| 276 | 3270.6 | 1175.91 | 1.01 | 1182.00 | 1175.91 | 1.13 | -0.59 | 1182.67 |
| 279 | 2508.0 | 712.34 | 0.71 | 716.57 | 712.34 | 0.74 | -0.20 | 716.80 |
| 280 | 3288.2 | 1286.36 | 1.14 | 1293.23 | 1286.36 | 1.40 | -1.38 | 1294.79 |
| 282 | 3199.7 | 1051.49 | 0.88 | 1056.76 | 1051.49 | 1.00 | -0.65 | 1057.50 |
| 283 | 2863.8 | 1082.86 | 1.10 | 1089.49 | 1082.86 | 1.13 | -0.13 | 1089.63 |
| 299 | 3539.8 | 1227.43 | 1.19 | 1234.59 | 1227.43 | 1.11 | 0.46 | 1234.07 |
| 300 | 2907.1 | 1168.34 | 0.96 | 1174.13 | 1168.34 | 1.00 | -0.21 | 1174.37 |
| 302 | 2847.4 | 1217.33 | 1.16 | 1224.26 | 1217.33 | 1.11 | 0.24 | 1223.99 |
| 304 | 3308.7 | 1255.71 | 1.29 | 1263.43 | 1255.71 | 1.36 | -0.38 | 1263.85 |
